# Utility of in vitro culture to the study of plant mitochondrial genome configuration and its dynamic features

**DOI:** 10.1007/s00122-012-1844-4

**Published:** 2012-03-18

**Authors:** Peibei Sun, Maria P. Arrieta-Montiel, Sally A. Mackenzie

**Affiliations:** Center for Plant Science Innovation, N305 Beadle Center, University of Nebraska, Lincoln, NE 68588-0660 USA

## Abstract

Recombination activity plays an important role in the heteroplasmic and stoichiometric variation of plant mitochondrial genomes. Recent studies show that the nuclear gene *MSH1* functions to suppress asymmetric recombination at 47 repeat pairs within the Arabidopsis mitochondrial genome. Two additional nuclear genes, *RECA3* and *OSB1*, have also been shown to participate in the control of mitochondrial DNA exchange in Arabidopsis. Here, we demonstrate that repeat-mediated de novo recombination is enhanced in Arabidopsis and tobacco mitochondrial genomes following passage through tissue culture, which conditions the *MSH1* and *RECA3* suppressions. The mitochondrial DNA changes arising through in vitro culture in tobacco were reversible by plant regeneration, with correspondingly restored *MSH1* transcript levels. For a growing number of plant species, mitochondrial genome sequence assembly has been complicated by insufficient information about recombinationally active repeat content. Our data suggest that passage through cell culture provides a rapid and effective means to decipher the dynamic features of a mitochondrial genome by comparative analysis of passaged and non-passaged mitochondrial DNA samples following next-generation sequencing and assembly.

## Introduction

The plant mitochondrial genome is organized into an unusual multipartite structure derived from high and low frequency DNA recombination between repeated sequences in the genome (Fauron et al. 1995). Large-sized (>1,000 bp) repeats participate in high-frequency reciprocal DNA exchange to subdivide the genome and facilitate inter-conversions between DNA molecules (Mackenzie and McIntosh 1999). Intermediate-sized (50–550 bp) repeats mediate low frequency asymmetric DNA exchange that results in accumulation of only one of the expected recombinant products (Shedge et al*.* 2007). Frequency of DNA exchange at these intermediate repeats appears to control the relative copy number of the recombinant forms within the genome.

In plants, the mitochondrial DNA population in vegetative tissues is organized into predominant and substoichiometric DNA configurations. Changes in relative abundance of these mitochondrial genomic forms, often occurring within a single plant generation, are referred to as substoichiometric shifting (SSS) (Arrieta-Montiel and Mackenzie 2011), a phenomenon first reported in maize (Small et al*.* 1987). The SSS process participates in the rapid generation of mitochondrial genome variation within a plant species (Davila et al*.* 2011), and appears to underlie reversible phenotypic transitions between cytoplasmic male sterile and male fertile plants within a population (Mackenzie 2011). Asymmetric recombination at intermediate-size repeats in mitochondria accounts for SSS activity (Arrieta-Montiel et al*.* 2009).

Three nuclear genes have been cloned and reported to participate in the control of plant mitochondrial recombination in Arabidopsis: *MSH1,* *RECA3* and *OSB1* (Abdelnoor et al*.* 2003; Shedge et al. 2007; Zaegel et al. 2006). Among these nuclear genes, *MSH1* appears to have the most profound effect on plant mitochondrial recombination surveillance and inherited plant phenotypic effects (Shedge et al. 2007; Sandhu et al. 2007). In Arabidopsis, mutation of *MSH1* elevates the mitochondrial DNA exchange activity at 47 repeat pairs ranging from 50 to 550 bp in size (Arrieta-Montiel et al*.* 2009; Davila et al*.* 2011). Although mitochondrial recombination and SSS activity can occur spontaneously at low frequency, disruption of nuclear genes *MSH1*, *RECA3* or *OSB1* enhances SSS frequency.

Earlier evidence of mitochondrial DNA polymorphisms arising in cultured cells is plentiful (Rode et al*.* 1987; Shirzadegan et al*.* 1989; Vitart et al*.* 1992; Hartmann et al*.* 2000). Most provocative among these reports is the observation of recombination events in tobacco that are reversible with plant regeneration, implying SSS activity under culture conditions (Kanazawa et al*.* 1994). We investigated the nature of mitochondrial genome changes under tissue culture conditions to assess the feasibility of capitalizing on this process for mitochondrial genome mapping. Here, we present evidence that tissue culture results in reduced expression of both *MSH1* and *RECA3*, together with enhanced recombination at intermediate repeats. This effect is reversible with plant regeneration, providing a useful system for the assembly of mitochondrial genome sequence into a more accurate physical map of the genome.

## Materials and methods

### Plant materials and growth conditions

Seeds from *Arabidopsis thaliana* Col-0 and *Nicotiana tabacum* cv. Xanthi were plated on 0.5× Murashige and Skoog media for germination. Antibiotic selection was performed by adding cefotaxime at 100 mg/ml to the germination plates. The seed plates were placed in a growth chamber at 24°C and 12-h day length provided by cool light fluorescent tubes, producing a photon flux density of 92–97 μmol m^−2^ s^−1^. Leaf segments of Arabidopsis (8–10 days old) and tobacco (14–16 days old) were transferred to callus-inducing medium for callus initiation, following the protocol of Encina et al*.* (2001). Callus-inducing medium for Arabidopsis consists of basic Murashige and Skoog (1962) supplemented with 1 mg L^−1^ 2,4-dichlorophenoxyacetic acid (2,4-D), 0.5 mg L^−1^ benzylaminopurine (BAP), 1 mg L^−1 ^1-Naphthyl acetic acid (NAA), 1 mg L^−1^ Indole-3-acetic acid (IAA), 3 % sucrose and 0.8 % washed agar (Sigma) with pH 5.7 ± 0.1. Callus-inducing medium for tobacco includes basic Murashige and Skoog inorganics and Gamborg’s B5 medium vitamins, supplemented with 0.25 mg L^−1^ benzylaminopurine (BAP), 1 mg L^−1^ 1-Naphthyl acetic acid (NAA), 3 % sucrose and 0.8 % Grade A agar (Sigma) with pH 5.7 ± 0.1. Callus cultures were maintained in dark at 24 °C.

In order to induce regeneration of tobacco shoots, 4-week-old tobacco callus was cultured on basic Murashige and Skoog inorganics and Gamborg’s B5 vitamins, supplemented with 1 mg L^−1^ benzylaminopurine (BAP), 0.1 mg L^−1^ 1-Naphthyl acetic acid (NAA) and 3 % sucrose. After formation of shoots, regenerated young seedlings were transferred to tobacco rooting medium with basic Murashige and Skoog inorganics, Gamborg’s B5 vitamins, 0.1 mg L^−1^ 1-Naphthyl acetic acid (NAA) and 3% sucrose. All media were solidified with 0.8% Grade A agar (Sigma) with pH 5.7 ± 0.1. Shooting and rooting occurred in a growth chamber at 24 °C and 18-h day length provided by cool light fluorescent tubes, producing a photon flux density of 92–97 μmol m^−2^ s^−1.^

### Total genomic DNA isolation, gel blot and PCR assays

1- to 4-week-old Arabidopsis and tobacco calli were sampled. Young leaves from Arabidopsis Col-0 and *msh1* mutant plants (Abdelnoor et al. 2003) and tobacco wildtype (Xanthi) plants served as control samples, which were grown in the growth chamber under the same condition as the growth chamber where seed plates were maintained. Total genomic DNA was isolated from calli and plants, according to the Li and Chory (1998) protocol. Total genomic DNA was digested with *Bam*HI (Arabidopsis) and *Cla*I (tobacco), and analyzed by DNA gel blot hybridization (Hybond-N, Amersham). Mitochondrial DNA repeats in Arabidopsis and tobacco mitochondrial genomes were PCR amplified, labeled with [α-^32^P] dCTP by random priming (Stratagene), and used as probes. Primers for PCR amplification of Arabidopsis and tobacco mitochondrial repeats are listed in Table 1. Primers for Arabidopsis mitochondrial repeats D and F were described previously (Arrieta-Montiel et al. 2009).

**Table 1:** Primers used in the study

Arabidopsis Repeat D-F	AGTGATCTGTTCATCTAACTCA
Arabidopsis Repeat D-R	TACTACTACCTCGTCCATTG
Arabidopsis Repeat F–F	CACGAGGAATGGAAAGAAACAT
Arabidopsis Repeat F-R	GCGCACAAACCACTCTAAAG
Tobacco Repeat A-F	TGGTAGTCGTGGTTGATTCGAGGAT
Tobacco Repeat A-R	TTAGGGGCGGAATCGAATGATTACG
Tobacco PCR-1F	GCGGCTACGAAGCAGTCAAG
Tobacco PCR-2R	TGAACACTGCTCTGCTGCATG
Tobacco PCR-3F	AGCGAAGAAAGCGGGCTTTG
Tobacco PCR-4R	ATTTCCCTCTATCAGGAACCCGCT
Tobacco Actin-F	GAACGGGAAATTGTCCGCGATGTT
Tobacco Actin-R	ATGGTAATGACCTGCCCATCTGGT
Arabidopsis Real-Ubquitin-F	CACCATTGACAACGTCAAGGCCAA
Arabidopsis Real-Ubquitin-R	CACGCAGACGCAAGACCAAATGAA
Arabidopsis Real-*MSH1*-F	TCATGCGTGTATGTGATGCGGAGA
Arabidopsis Real-*MSH1*-R	ACTTGACCCTTGCAGTCCTTCCTT
Arabidopsis Real-*RECA3*-F	ATCTAACATGCATTTCCCGCACGC
Arabidopsis Real-*RECA3*-R	TGGACGCAGACATTGAGACCACTT
Arabidopsis Real-*OSB1*-F	ACGATTGGTGGGACAACAGGAGAA
Arabidopsis Real-*OSB1*-R	TCTGAGCAAAGCCAGAGAGCTTCA
Tobacco Real-Ubquitin-F	TTTGCACCTTGTGCTTCGTCTTCG
Tobacco Real-Ubquitin-R	CCATCTTCCAATTGCTTTCCCGCA
Tobacco Real-*MSH1*-F	TGATGGATCCTACTTGGGTGGCAA
Tobacco Real-*MSH1*-R	ACCTTTCCATGGCGACTCCATATC

Total genomic DNAs from tobacco regenerants at three different growth stages were assayed by three-primer competitive PCR. Primers (Table 1) were designed to assay each environment flanking Repeat A. Actin was used as internal control.

### RNA isolation and real-time quantitative PCR analysis

Total RNA was extracted from Arabidopsis and tobacco callus, and wildtype and regenerated plants, with TRIzol (Invitrogen) and purified with RNeasy (Qiagen). Purified RNA was used to synthesize first-strand cDNA (SuperScript III First-Strand Synthesis SuperMix for qRT-PCR; Invitrogen). Equal amounts of cDNA were used for quantitative PCR with SYBR GreenER for iCycler (Invitrogen). Quantitative PCR analysis used iCycler iQ software (version 3.1, Bio-Rad). The ubiquitin gene was used as internal standard in gene expression analysis (Arrieta-Montiel et al. 2009). Fold change for *MSH1,* *RECA3* or *OSB1* expression in each sample, compared to wildtype, was calculated as $2^{{ - \Updelta \Updelta C_{\text{t}} }}$ (ΔΔ*C*_t_ = Δ*C*_tS_ − Δ*C*_tWT_, Δ*C*_tS_ = *C*_t*MSH1*_ − *C*_tUbq_, Δ*C*_tWT_ = *C*_t*MSH1*_ − *C*_tUbq_). Primers for RT-PCR analysis of ubiquitin, *MSH1*, *RECA3* and *OSB1* in Arabidopsis and ubiquitin and *MSH1* in tobacco are included in Table 1.

## Results

### Mitochondrial recombination increased markedly at very early stages of Arabidopsis and tobacco callus culture

**Fig. 1:**
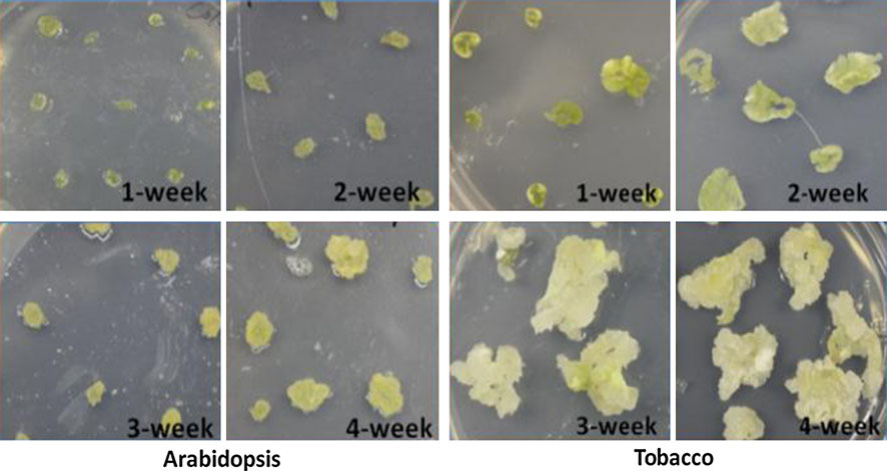
1- to 4-week-old Arabidopsis and tobacco calli on callus-inducing medium. Calli were formed out of leaf explants beginning week 1. By week 3, almost all the leaf explants were calli. 1- and 2-week photographs showed the leaf explants together with calli

**Fig. 2:**
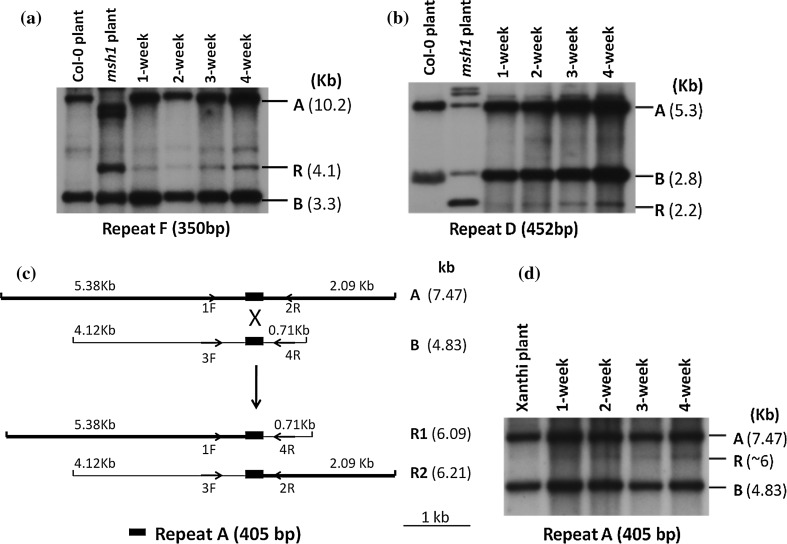
Repeat-mediated recombination in Arabidopsis and tobacco callus cultures. DNA gel blot analysis shows changes in mitochondrial DNA configurations, with *A* and *B* designating parental configurations and *R* the recombinant form. The indicated repeats were the only probes used. Different stages of callus samples represented repeated experimental results. **a** Analysis of recombination at Repeat F in 1- to 4-week-old Arabidopsis callus. Col-0 and *msh1* mutant plant tissues served as controls, and DNA was digested with *BamH*1. **b** Analysis of recombination at Repeat D in 1- to 4-week-old Arabidopsis callus. Col-0 and *msh1* mutant plant tissues served as controls, and DNA was digested with *BamH*1. **c** *Cla*I restriction map of the parental forms (*A* and *B*) and the predicted recombinant forms (*R*1 and *R*2) in tobacco mitochondria. *1F*, *2R* and *4R* are primers used for three-primer competitive PCR. **d** Evidence of mitochondrial DNA recombination at Repeat A in 1- to 4-week-old tobacco callus. Xanthi plant tissue serves as a control, and DNA was digested with *Cla*I

Initiation of callus formation could be detected 1 week following placement of Arabidopsis and tobacco leaf segments on callus-inducing medium, with mature friable callus formed after 4 weeks (Fig. 1). Total genomic DNA extracted from 1- to 4-week-old calli was digested with *Bam*HI (Arabidopsis) or *Cla*I (tobacco) for mitochondrial genome analysis. In Arabidopsis, repeat-mediated recombination was detected in 1-week-old cultures, with the level of recombination increasing over time. The 4-week-old cultured tissues displayed the same 4.1-kb recombinant form at Repeat F that is observed in the *msh1* mutant (Arrieta-Montiel et al. 2009), while both parental forms were also retained (Fig. 2a). Recombination at Repeat D gave the predicted 2.2-kb recombinant molecule in callus culture, again increasing with time (Fig. 2a). In tobacco callus, recombination at Repeat A was evident in the predicted recombinant 6-kb form (Fig. 2c), which is observed only at very low levels in the wildtype plant.

### *MSH1* and *RECA3,* but not *OSB1,* expression levels are modulated under tissue culture conditions in Arabidopsis

**Fig. 3:**
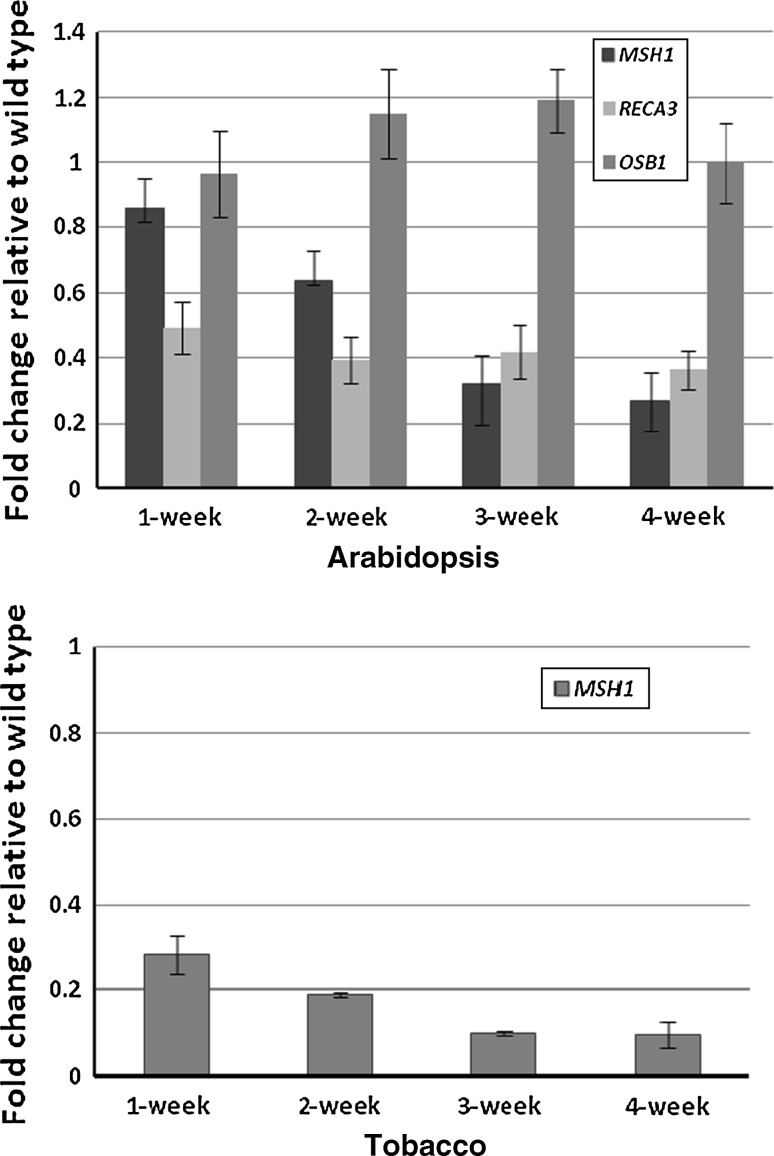
*MSH1*, *RECA3* and *OSB1* gene expression changes in 1- to 4-week-old Arabidopsis and tobacco calli. Gene expression is interpreted as the threshold cycle number (*C*_t_) and normalized as fold change, compared with wildtype control plants set at 100%. Ubiquitin serves as an internal standard. The final results were the average of three biological replicates

Real-time PCR analysis in Arabidopsis and tobacco callus showed down-regulation of *MSH1* expression relative to wildtype (Col-0 and Xanthi) plant samples (Fig. 3). In both Arabidopsis and tobacco, the decrease in expression was more pronounced with age of callus. *RECA3* expression was also down-regulated in Arabidopsis, but *OSB1* transcript levels did not change under tissue culture conditions. We suggest that the mitochondrial genomic rearrangements observed are the likely consequence of altered *MSH1* and *RECA3* expression.

### *MSH1* expression is correlated with SSS activity during tobacco plant regeneration

**Fig. 4:**
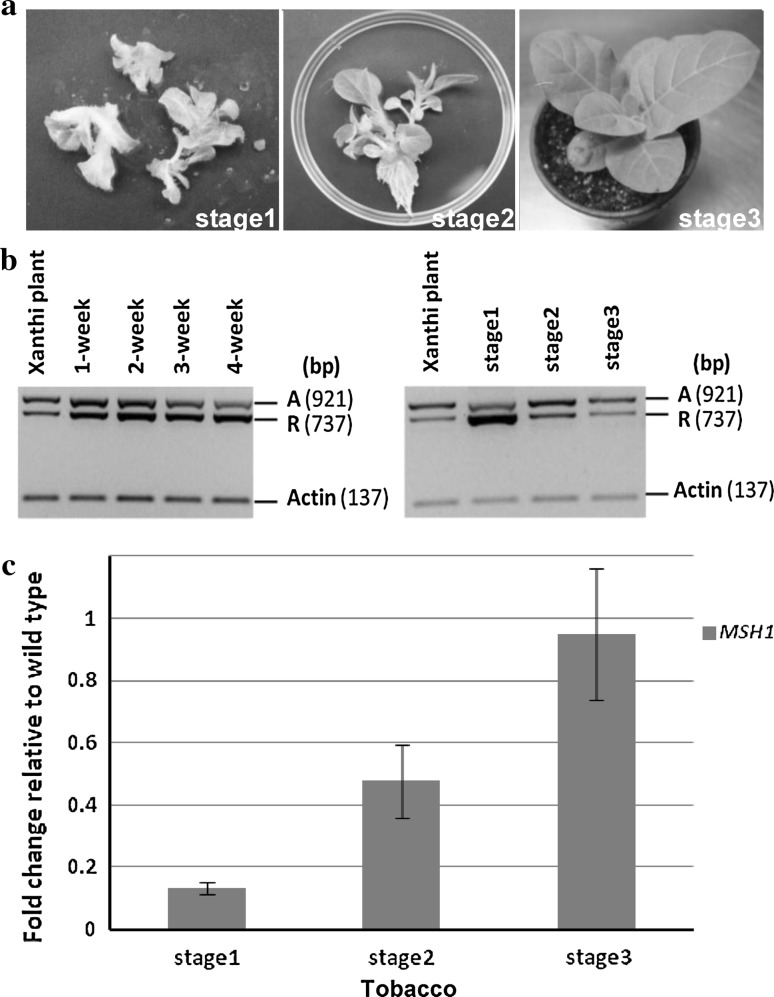
Mitochondrial genome configuration and associated *MSH1* expression changes during tobacco regeneration. **a** Tobacco regenerants at three different growth stages from 4-week-old callus. **b** Substoichiometric shifting detected by PCR in experiments involving 1- to 4-week-old tobacco calli and regeneration from 4-week-old tobacco calli at three stages. 1F, 2R and 4R are the primers used in three-primer competitive PCR. Actin is used as an internal standard. *A* designates the parental band, while *R* designates the amplified recombinant band. Different stages of callus samples and regenerants represented repeated experimental results. **c** *MSH1* gene expression fold changes by real-time PCR in regenerated tobacco at three stages. Xanthi plants serve as a control and ubiquitin as an internal standard. Results shown are the average of three biological replicates

During tobacco regeneration from 4-week-old callus, three stages were investigated (Fig. 4a): Stage 1, 1 month following transfer of 4-week-old callus to shooting medium; Stage 2, 2 months following callus transfer, when roots emerge in rooting medium; and Stage 3, 1 week following transfer of the young seedling to potting mix. Genomic DNA was extracted from the different growth stages, and three-primer competitive PCR analysis (Fig. 4b) allowed resolution of changes in relative stoichiometries for parental and recombinant forms during the regeneration process. Recombinant forms were predominant in callus, and Stage 1 reversed the trend back toward substoichiometric levels in the subsequent regeneration stages. Recombinant and parental forms were confirmed by DNA sequencing. Similarly, down-regulation of *MSH1* expression in Stage 1 was gradually reversed during regeneration, reaching normal *MSH1* transcript levels in the regenerated plant (Fig. 4c).

## Discussion

Over the past 20 years, mitochondrial genome rearrangements were often reported to occur in plant cells grown in vitro (Cloutier and Landry 1994). It was generally thought that these rearrangements were the consequence of extended culture periods. Evidence presented here of mitochondrial SSS activity within the first week of callus culture suggests that the rearrangement activity is non-random and initiates immediately. Previous studies of the *msh1* mutant in Arabidopsis, involving 47 mitochondrial repeats that are enhanced in DNA exchange activity (Arrieta-Montiel et al. 2009; Davila et al. 2011), permitted assay of SSS activity under cell culture conditions. These earlier studies showed that an “early” generation *msh1* mutant produces mitochondrial genome rearrangements at lower frequency that are reversible following re-introduction of the *MSH1* gene (Davila et al. 2011). The “advanced” generation *msh1* mutants produced more extensive rearrangements that were less readily reversible and could become fixed in the mitochondrial population. Combining the *msh1* and *recA3* mutations resulted in the most extensive mitochondrial genome rearrangements (Shedge et al*.* 2007). In this study, we have shown that down-regulation of both *MSH1* and *RECA3* occurs under cell culture conditions, suggesting that conditions are appropriate for rapid and extensive mitochondrial genome changes.

A question raised by this study is whether plant regeneration would be facilitated if the mitochondrial genome were stably maintained during in vitro culture. Tobacco, where plants can be readily regenerated from callus, has been reported to display a highly reversible mitochondrial rearrangement process (Kanazawa et al*.* 1994). We also observed reversibility of mitochondrial genome rearrangement in tobacco upon regeneration from callus. Might this reversibility be a factor in the plant’s amenability to regeneration? There appear to be several plant developmental implications of the *msh1 recA3* double mutation (Shedge et al. 2007, 2010), suggesting that this type of gene expression change can have dramatic implications for plant growth.

Because the plant mitochondrial genome is characterized by numerous recombinational repeats, assembly of an intact genome sequence, particularly using deep sequence reads of relatively small size, can be extremely difficult. Sequence assembly without the availability of information from substoichiometric forms provides an incomplete picture of the genome, so that intra-specific mitochondrial comparative studies can imply far more extensive genomic variation than is actually present. Information about substoichiometric forms can allow one to deduce the inter-convertibility of related mitochondrial configurations. In many plant species, particularly those being investigated for ecological studies, little mitochondrial genome information is currently available and details of intra-specific mitochondrial relationships may be of crucial importance. In these cases, implementing callus culture for mitochondrial genome analysis may be valuable. Comparative assembly of the callus culture-derived mitochondrial genome sequence and intact plant-derived form would reveal recombinational repeats and substoichiometric forms. This type of information is valuable for subsequent ecotype comparisons and for developing an understanding of evolutionary trends within a species (Davila et al*.* 2011).
